# Supplementary material for: Synthesis, Characterization and Potential Antimicrobial Activity of Selenium Nanoparticles Stabilized with Cetyltrimethylammonium Chloride
Source: Nanomaterials (Basel). 2023 Dec 13;13(24):3128. doi: 10.3390/nano13243128 (PMC10746028; doi:10.3390/nano13243128)
Supplement: Supplementary file 1 [file nanomaterials-13-03128-s001.zip › nanomaterials-2610108-supplementary.pdf]

Supplementary

S1. Preparation of NaOH solutions

Table S1. NaOH volumes with corresponding pH values

NaOH x (mL)	pH
0	1.81 ± 0.05
10	2.21 ± 0.05
20	3.29 ± 0.05
30	4.56 ± 0.05
40	5.72 ± 0.05
50	6.8 ± 0.05
60	7.96 ± 0.05
70	9.15 ± 0.05
80	10.38 ± 0.05
90	11.58 ± 0.05
100	11.98 ± 0.05

S2. The main stages of preparation of nutrient media and cultivation of *Escherichia coli, Micrococcus luteus and Mucor*

For *Escherichia coli* culture, 17.55 g of Endo nutritional medium was combined with 450 ml of distilled water. Endo medium was made by adding 1ml of sterile molten meat-peptone agar (Reagent grade, Stavreakhim, Russia) and 1ml of a saturated alcoholic solution of basic fuchsin (Analytical grade, MiniMed, Russia) to 100ml of sterile molten meat-peptone agar (Reagent grade, Stavreakhim, Russia). The solution was tinted with a 10% aqueous solution of sodium sulfite (Ultrapure, Stavreakhim, Russia). After that, the mixture was mixed and put onto Petri plates. The mixture was cooked for 2 minutes, or until the agar melted completely.

The medium was placed in sterile vials after filtration through a cotton gauze filter, after which the medium was autoclaved at 121°C for 15 minutes, then cooled to 45–50°C and poured into sterile Petri dishes in 20–25 ml portions. Prior to reseeding the culture, the medium was dried for 40-45 minutes after solidification.

To cultivate *Micrococcus luteus*, 16.24 g of meat peptone agar nutrient medium was mixed with 450 ml of distilled water. To prepare meat-peptone agar, the following method was used: 15-20 g of agar (analytical grade, Merck, Germany) was added to 1 liter of meat-peptone broth. The mixture was heated over low heat until completely dissolved and the volume of liquid was brought to the original volume with distilled water and the solution was filtered through a gauze filter. The heated medium was placed in glass flasks and sterilized for 20 minutes in an autoclave at 150°C.

After boiling for 2 minutes to completely melt the agar, the liquid was filtered through a cotton-gauze filter and poured into sterile vials. The medium was autoclaved at 121°C for 15 minutes, then cooled to 45-50°C and poured in portions of 20-25 ml into sterile Petri dishes. Before re-seeding the culture, the medium was dried for 40-45 minutes after hardening.

For the *Mucor* culture, 16.24 g of meat peptone agar nutrient medium was mixed with 450 ml of distilled water and heated for 2 minutes or until the agar was completely dissolved. The resulting mixture was filtered through a cotton gauze filter and placed in sterile containers, which were then autoclaved at 121°C for 15 minutes for disinfection. The medium was applied to sterile Petri dishes in a volume of 20-25 ml after cooling to 45-50°C and allowed to harden for 40-45 minutes. The culture was then introduced and allowed to flourish.

The microbial loop was heated on an alcohol lamp, the Petri dish containing cultures of *Escherichia coli*, *Micrococcus luteus* and *Mucor* was carefully opened, one colony was selected from a separate area using a cooled microbiological loop, the culture was evenly distributed over the surface of the Petri dish with sterile nutrient material, the inoculated culture in the dish The petri was closed, after which the microbiological loop was disinfected with the flame of an alcohol lamp.

S3. Determination of the concentration of Se NPs

The concentration of Se NPs was calculated theoretically. For this purpose, preliminary experiments were carried out, as a result of which the reaction yield (η, %) was determined.

$$\eta = m_{practical}(Se) / m_{theoretical}(Se) \quad (S1)$$

Thus, the molar concentration of Se NPs was calculated using the formula:

$$C_M(Se) = m_{practical}(Se) * V * M \quad (S2)$$

where C_M (Se) – molar concentration, $m_{theoretical}$ (Se) – mass of substance, M – molar mass, V – volume of solution.

Based on formula (S1):

$$m_{practical}(Se) = m_{theoretical}(Se) * \eta \quad (S3)$$

Then:

$$C_M(Se) = m_{practical}(Se) * V * M = m_{theoretical}(Se) * V * M * \eta \quad (S4)$$

Taking into account the reaction equation for the production of Se NPs, given in the text of the article, we obtain that:

$$m_{sample}(H_2SeO_3) / M(H_2SeO_3) = m_{theoretical}(Se) / M(Se) \quad (S5)$$

in this connection:

$$m_{theoretical}(Se) = m_{sample}(H_2SeO_3) * M(Se) / M(H_2SeO_3) \quad (S6)$$

Based in formula (S4):

$$C_M(Se) = m_{practical}(Se) * V * M = m_{theoretical}(Se) * V * M * \eta =$$

$$m_{sample}(H_2SeO_3) * M(Se) * V * M * \eta / M(H_2SeO_3)$$
